# Multimodal imaging features of high-grade endometrial stromal sarcoma with pulmonary and nodal metastases: a case report and literature review

**DOI:** 10.3389/fmed.2025.1676847

**Published:** 2025-09-02

**Authors:** Huayun Tan, Tingting Zhang

**Affiliations:** Obstetrics Medical Center, Weifang People’s Hospital, Shandong Second Medical University, Weifang, Shandong, China

**Keywords:** endometrial stromal sarcoma, multimodal imaging, neoplasm metastasis, diffusion magnetic resonance imaging, diagnostic imaging

## Abstract

**Background:**

High-grade endometrial stromal sarcoma (ESS) is an aggressive tumor that poses significant diagnostic challenges, particularly when associated with multifocal metastases. Multimodal imaging, including ultrasound, computed tomography (CT), and magnetic resonance imaging (MRI), plays a critical role in diagnosis.

**Case presentation:**

A 51-year-old woman presented with abnormal vaginal bleeding and pelvic pain. Ultrasound revealed an 8 × 12 × 17 cm heterogeneous mass located in the uterine body and cervix. CT scans showed metastatic pelvic lymphadenopathy and pulmonary nodules. MRI demonstrated a T2-hyperintense lesion with myometrial and cervical invasion, along with restricted diffusion on diffusion-weighted imaging (DWI) and apparent diffusion coefficient (ADC) mapping. Pathological analysis confirmed high-grade ESS.

**Results:**

Multimodal imaging successfully differentiated high-grade ESS from benign uterine lesions by identifying aggressive features such as ill-defined margins on ultrasound, areas of necrosis on CT, and restricted diffusion on MRI. The patient was diagnosed with stage IVB (T3bN1M1) high-grade ESS, due to pelvic lymph node involvement and multiple pulmonary metastases.

**Conclusion:**

High-grade ESS is prone to distant metastasis. MRI with DWI and ADC mapping is crucial for assessing tumor cellularity and local invasion, while CT is effective for detecting metastases. Future staging protocols should integrate contrast-enhanced MRI and positron emission tomography–computed tomography (PET–CT) for comprehensive assessment.

## 1 Introduction

Endometrial stromal sarcoma (ESS) is a rare uterine malignancy, accounting for less than 0.2% of all uterine cancers. It is classified into low-grade and high-grade subtypes, with high-grade ESS being more aggressive and associated with a poorer prognosis (1, 2). ESS often presents with non-specific symptoms such as abnormal bleeding and pelvic pain, which overlap with benign conditions such as uterine leiomyomas (3).

Imaging techniques, including ultrasound, computed tomography (CT), and magnetic resonance imaging (MRI), play a critical role in the diagnosis of ESS. MRI, particularly with diffusion-weighted imaging (DWI), is crucial for differentiating ESS from benign uterine masses by highlighting differences in cellularity. DWI helps identify areas of high cellularity through low apparent diffusion coefficient (ADC) values, while contrast-enhanced MRI can reveal necrosis and hemorrhage (2, 4). However, distinguishing high-grade ESS from low-grade ESS remains challenging, as high-grade lesions tend to show more extensive necrosis, hemorrhage, and the potential for distant metastasis (5).

Single-photon emission computed tomography/computed tomography (SPECT/CT) can help in detecting unusual sites of metastasis, such as bone, which, although rare in high-grade ESS, may be overlooked by standard imaging. A recent case report demonstrated that SPECT/CT successfully identified bone involvement in high-grade ESS, which led to a change in clinical management (6). PET/CT, particularly fluorine-18 fluorodeoxyglucose positron emission tomography/computed tomography (^18^F-FDG PET/CT), provides combined metabolic and anatomical data, improving staging accuracy, revealing distant or occult metastases when conventional imaging is inconclusive, and aiding in treatment response assessment during follow-up (7, 8).

In this study, we present the case of a 51-year-old female with a mass in the uterine body and cervix, diagnosed as high-grade ESS with pelvic lymph node involvement and multiple pulmonary metastases. This case underscores the importance of multimodal imaging, including ultrasound, CT, and MRI in accurately assessing disease extent and guiding both the diagnosis and management of ESS.

## 2 Case presentation

We present the case of a 51-year-old woman who presented with a 1-year history of abnormal vaginal discharge, intermittent vaginal bleeding, and lower abdominal pain. She had no significant past medical history or family history of malignancies. An ultrasound performed at a previous hospital suggested an abnormal uterine mass, prompting further evaluation at our institution. Serum tumor marker CA-125 was elevated at 102.0 U/mL (normal reference range: 0–25 U/mL).

**Figure 1:**
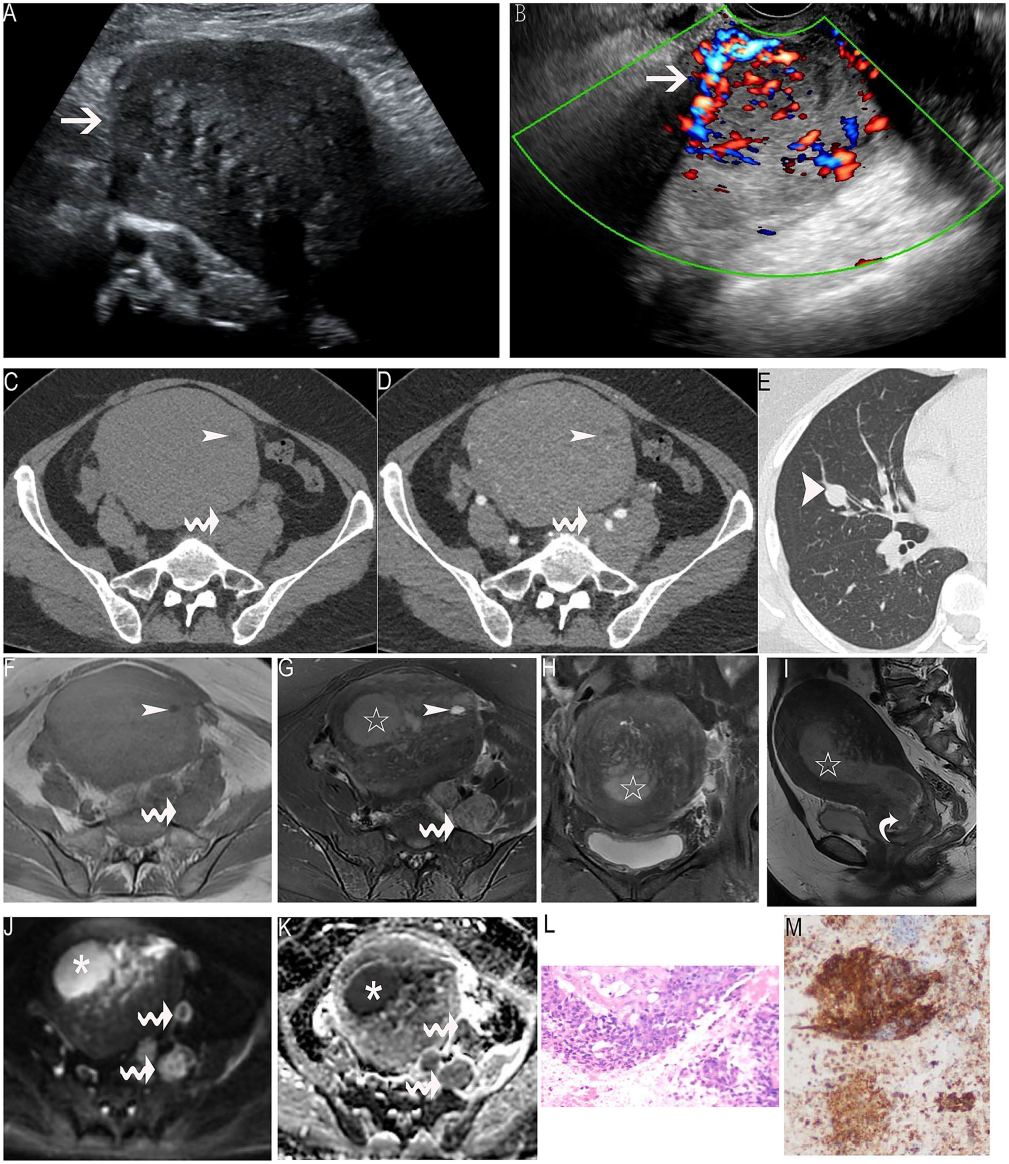
Imaging and histopathological features of high-grade endometrial stromal sarcoma (ESS). **(A)** A mass measuring 8 × 12 × 17 cm identified in the uterine body and cervix, exhibiting increased echogenicity, irregular shape, and heterogeneous internal echoes with cystic low-echo areas (arrow). **(B)** Color Doppler ultrasound revealing abundant mixed blood flow within and around the mass (arrow). **(C)** Pelvic axial computed tomography (CT) showing an enlarged uterine body with an irregular soft tissue mass in the uterine cavity and the cervix. The plain CT value was 47 HU, with cystic low-attenuation areas (thin arrow). **(D)** Pelvic axial CT during the arterial phase demonstrating multiple enhancing blood vessels and cystic areas within the mass (thin arrow). Metastatic lymph nodes were noted near the iliac vessels, which were more prominent on the left (wavy arrows). **(E)** Chest axial CT revealing multiple pulmonary nodules, with the largest located in the right middle lobe (thick arrow). **(F)** Pelvic axial magnetic resonance imaging (MRI) showing a large uterine mass with cystic areas exhibiting hypointensity on T1-weighted images (thin arrow). **(G)** Pelvic axial MRI showing the large uterine mass with cystic areas exhibiting hyperintensity on T2-weighted images (thin arrow). **(H)** Coronal T2-weighted MRI showing a high-signal lesion in the endometrial cavity (star-shaped), extending into the myometrium with multiple low-signal linear areas. **(I)** Sagittal T2-weighted MRI showing a high-signal tumor in the endometrial cavity (star-shaped), with downward invasion into the cervix (curved arrow). **(J)** Diffusion-weighted imaging (DWI) showing high signal intensity in the solid portion of the tumor (*) and the left-sided iliac lymph nodes (wavy arrows). **(K)** Apparent diffusion coefficient (ADC) map showing restricted diffusion with low signal intensity in the solid portion of the tumor (*) and the left-sided iliac lymph nodes (wavy arrows). **(L)** Pathological image showing extensive necrosis with nests of atypical cells. **(M)** Immunohistochemistry demonstrating positivity for CD10, thereby confirming the diagnosis of high-grade ESS.

Transvaginal pelvic ultrasound showed an enlarged uterus with abnormal cervical morphology and a poorly defined endometrium. A mass measuring 8 × 12 × 17 cm was identified involving the uterine body and cervix, characterized by increased echogenicity, irregular shape, and heterogeneous internal echoes with cystic low-echo areas (Figure 1A, arrow). Color Doppler imaging revealed abundant mixed blood flow within and surrounding the mass (Figure 1B, arrow).

Pelvic axial CT showed an enlarged uterine body with an irregular soft tissue mass in the uterine cavity and cervix. The plain CT value was 47 HU, with cystic low-attenuation areas (Figure 1C, thin arrow). During the arterial phase, multiple enhancing blood vessels and cystic regions were observed (Figure 1D, thin arrow). Metastatic lymph nodes were noted near the iliac vessels, more prominent on the left (Figures 1C,D, wavy arrows). Chest axial CT lung window revealed multiple pulmonary nodules, with the largest located in the right middle lobe (Figure 1E, thick arrow).

Pelvic axial MRI revealed a large mass in the uterine cavity, with cystic areas hypointense on T1-weighted images and hyperintense on T2-weighted images (Figures 1F,G, thin arrows). The solid portion of the tumor demonstrated a signal intensity similar to the surrounding myometrium on T1-weighted images and higher intensity on T2-weighted images (Figures 1F,G). Left-sided iliac lymph node metastasis was identified (Figures 1F,G, wavy arrows). Coronal (Figure 1H) and sagittal T2-weighted images (Figure 1I) showed a high-signal lesion in the endometrial cavity (star-shaped), extending into the surrounding myometrium with multiple low-signal linear areas. The tumor invaded the cervix (Figure 1I, curved arrow). DWI (Figure 1J) showed high signal intensity in the solid portion of the tumor (*), with low signal intensity on the ADC map (*), indicating restricted diffusion (Figure 1K). Similar restricted diffusion was observed in the left-sided iliac lymph nodes (Figures 1J,K, wavy arrows).

Following diagnostic curettage and cervical biopsy, approximately 20 g of friable tissue was obtained from the cervical canal and 15 g from the lower uterine segment. Pathological examination revealed extensive necrosis with nests of atypical cells (Figure 1L). Immunohistochemistry showed positivity for CD10 and Ki67 (60% positivity), confirming the diagnosis of high-grade ESS (Figure 1M).

Based on imaging and histological findings, the tumor was staged as T3bN1M1, corresponding to stage IVB high-grade ESS. Given the presence of distant metastasis, the patient was no longer considered a candidate for surgery. Instead, she was recommended to undergo chemotherapy with doxorubicin and radiation therapy to alleviate symptoms. She received systemic chemotherapy with doxorubicin at 60 mg/m^2^ every 3 weeks for 4 cycles, followed by palliative external beam radiotherapy to the pelvis, delivered as 30 Gy in 10 fractions over 2 weeks. Follow-up by phone revealed that the patient remains alive, but disease control is suboptimal.

## 3 Discussion

In this study, we presented the case of a 51-year-old woman diagnosed with high-grade ESS. The diagnosis was done through advanced imaging and pathological evaluation. The case underscores the critical role of imaging techniques, including ultrasound, CT, and MRI, in differentiating ESS from other uterine tumors, such as benign leiomyomas and endometrial carcinoma. ESS can be differentiated from other uterine tumors, such as benign leiomyomas and endometrial carcinoma, using advanced imaging. On ultrasound, ESS presents as a mass with ill-defined margins and heterogeneous echogenicity, possibly involving the myometrium or protruding into the endometrial cavity with nodular extensions (9). In this case, the endometrium is unclear, and the tumor has invaded the serosal layer. In contrast, leiomyomas are usually well-defined, hypoechoic masses with peripheral rims. On MRI, ESS shows extensive myometrial invasion, with cystic high-signal necrosis and low-signal intensity bands on T2-weighted images, indicating preserved myometrial fibers, a feature less common in endometrial carcinoma (4).

Compared with previously reported cases, this patient presented with both pelvic lymph node metastasis and multiple pulmonary nodules at initial diagnosis, highlighting a more advanced disease stage and emphasizing the importance of early multimodal imaging for detection (1–14). A mass protruding from the myometrium into the uterine cavity, or an ill-defined uterine mass with mixed cystic and solid components, is characteristic of endometrial ESS. Color Doppler ultrasound showed irregular blood vessels with a low resistance index (RI), indicating malignant potential (9). CT imaging, while limited in resolution, is still valuable for identifying internal cystic necrosis areas, areas of enhanced vascularity, and metastasis. This technique can easily detect pelvic lymph node involvement and distant lung metastases, which are common in high-grade ESS and indicate its aggressive nature (5). Unfortunately, no further PET/CT examination was performed to search for additional metastatic lesions.

High-grade ESS is an aggressive uterine malignancy with limited effective treatment options. Surgery remains the mainstay for localized disease, but advanced or recurrent cases often respond poorly to conventional chemotherapy. Agents such as doxorubicin and ifosfamide offer modest benefits, while newer regimens such as gemcitabine–docetaxel show some activity but with considerable toxicity. Hormonal therapy is rarely effective due to low receptor expression in high-grade tumors. No standard systemic regimen has been established, and targeted therapies remain investigational (14). In the present case, the presence of distant pulmonary metastases precluded surgical intervention. The response to chemotherapy and radiotherapy was suboptimal, highlighting the importance of comprehensive multimodal imaging in guiding prognosis and therapeutic planning.

On MRI, ESS shows extensive myometrial involvement with characteristic low-signal intensity bands on T2-weighted images, particularly in low-grade ESS. High-grade ESS may have fewer of these bands due to more aggressive growth. DWI reveals restricted diffusion in high-grade ESS, seen as high signal intensity. Moreover, ADC values are lower in high-grade ESS, indicating increased cellularity. High-grade ESS also shows more extensive necrosis, hemorrhage, and feather-like enhancement, which are highly effective in distinguishing it from low-grade ESS. Both grades present with T2 low-signal bands, marginal nodules, and worm-like myometrial nodules. DWI and ADC are useful for assessing tumor aggressiveness and guiding preoperative planning. Diffusion-weighted MRI and contrast enhancement, particularly feather-like enhancement, aid in differentiating between high-grade and low-grade ESS (1, 3).

In this study, MRI contrast enhancement was not performed, which limited the ability to fully assess the vascular characteristics and enhance the differentiation between high-grade and low-grade ESS. The high blood flow observed on ultrasound and significant vascular enhancement on CT suggest the rich blood supply typical of high-grade ESS, but contrast-enhanced MRI could have provided more detailed information on tumor vascularity and further confirmed the tumor’s aggressiveness (4, 10, 11). Future studies should involve MRI with contrast enhancement, particularly to assess feather-like enhancement, which is a key feature in distinguishing high-grade ESS from low-grade ESS.

High-grade ESS frequently metastasizes to the lungs and pelvic or para-aortic lymph nodes, while less common sites include the liver, bones, peritoneum, adrenal glands, and heart (6, 7, 14). Therefore, multimodal imaging, including ultrasound, CT, MRI, PET/CT, and SPECT/CT, is essential for evaluating both local invasion and distant metastases to achieve accurate staging and guide effective management. Additionally, incorporating PET/CT scans could help identify distant metastases and provide a comprehensive view of the tumor’s extent, further guiding treatment decisions and efficacy evaluation (5).

## 4 Conclusion

High-grade ESS is a rare but highly aggressive malignancy with a strong tendency for local invasion and distant metastasis. Early and accurate diagnosis relies heavily on a multimodal imaging strategy. Ultrasound, CT, and particularly MRI with DWI and ADC mapping are indispensable tools for evaluating tumor characteristics and planning treatment. Contrast-enhanced MRI and PET/CT should be considered essential components of the diagnostic workup to optimize staging, therapeutic decisions, and prognosis in patients with suspected high-grade ESS.

## Data Availability

All data presented in this article are contained within the manuscript itself. No publicly available datasets were analyzed in this study.
